# Effects of Omega-3 PUFAs on lipid profiles and antioxidant response in depressed adolescents: A metabolomic and lipidomic study

**DOI:** 10.1016/j.redox.2025.103617

**Published:** 2025-03-25

**Authors:** Jinfeng Wang, Shuhui Li, Dandan Wang, Yan Gao, Qian Wang, Tianqi Wang, Guanghai Wang, Daihui Peng, Yi Qiao, Jiansong Zhou, Lei Feng, Xiaowen Hu, Chunling Wan

**Affiliations:** a Bio-X Institutes, Key Laboratory for the Genetics of Developmental and Neuropsychiatric Disorders, Ministry of Education, Shanghai Jiao Tong University, Shanghai, China; b Department of Developmental and Behavioral Pediatrics, Pediatric Translational Medicine Institute, Shanghai Children's Medical Center, School of Medicine, Shanghai Jiao Tong University, Shanghai, China; c Shanghai Mental Health Center, Shanghai Jiao Tong University School of Medicine, Shanghai, China; d Department of Psychiatry, National Clinical Research Center for Mental Disorders, and National Center for Mental Disorders, The Second Xiangya Hospital of Central South University, Changsha, Hunan, China; e Instrumental Analysis Center, Shanghai Jiao Tong University, Shanghai, China

**Keywords:** Adolescent depression, ω3 PUFA, Metabolome, Lipidome, Lipid oxidation, Personalized therapy

## Abstract

Adolescent depression is a significant global health challenge, with many patients responding inadequately to antidepressant treatments. Omega-3 polyunsaturated fatty acids (ω3 PUFAs) have been proposed as a potential adjunctive treatment, but their precise mechanisms remain poorly understood. This study aimed to explore the mechanisms through which ω3 PUFAs exert their antidepressant effects and to identify potential biomarkers for their therapeutic response. A comprehensive assessment of plasma metabolomic and erythrocyte membrane lipidomic was performed on 51 depressed adolescents who were randomly assigned to received either ω3 PUFAs plus paroxetine (n = 27) or paroxetine alone (n = 24) for 12 weeks. Following ω3 PUFA supplementation, phospholipid metabolism emerged as the most significantly altered pathway. ω3 PUFAs markedly influenced the composition of membrane fatty acids, significantly increasing the ω3 PUFA content, decreasing the ω6/ω3 PUFA ratio, and increasing membrane fluidity. Notably, ω3 PUFAs reduced lipid peroxidation in both plasma and cell membranes while enhancing antioxidant capacity in the membranes. Moreover, alterations in phospholipids and membrane function were significantly correlated with improvements in depressive symptoms and cognitive function. Importantly, ω3 PUFA supplementation resulted in greater improvement in clinical symptoms compared to the non-supplemented group exclusively in the subgroup with high baseline oxidative damage levels. This study suggests that ω3 PUFAs promoted phospholipid integration and alleviated oxidative stress, which may account for their antidepressant effects. Lipid oxidation biomarkers could help identify patients likely to benefit from ω3 PUFA supplementation. These findings advance our understanding of the mechanism and clinical application of ω3 PUFAs in treating adolescent depression.

## Highlights

• Metabolome and lipidome are used to explore the therapeutic mechanisms of ω3 PUFAs.
• ω3 PUFAs alter phospholipid metabolism and membrane fatty acid composition.
• Membrane fluidity and serotonin levels increase after ω3 PUFA supplementation.
• Reduction in lipid oxidation is a primary antidepressant mechanism of ω3 PUFAs.
• Lipid oxidation levels predict response to ω3 PUFA treatment in adolescent depression.

## Graphical abstract

**Figure:**
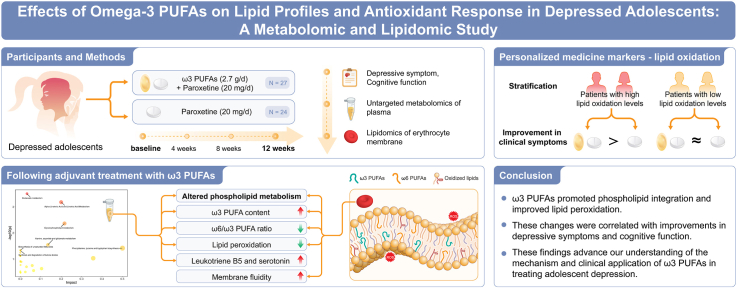

## 1 Introduction

Depression is one of the most prevalent mental health disorders in adolescents, with a lifetime prevalence rate of 19% among teenagers [1,2]. Adolescence is a period with rapid developments in cognition and emotion. Depression permanently impairs the psychological functioning and quality of life, making it the fourth leading cause of disease burden among individuals aged 10–24 years [3]. Consequently, prevention and early intervention for depression in adolescents are paramount public health priorities. Although antidepressants represent the primary pharmacological treatment for adolescents with depression, they often exhibit limited efficacy compared to placebo, not to mention that many patients experience treatment resistance or severe adverse effects, including mania and suicidal ideation [[4], [5], [6]].

Omega-3 polyunsaturated fatty acids (ω3 PUFAs), primarily docosahexaenoic acid (DHA) and eicosapentaenoic acid (EPA), play crucial roles in the development and function of the brain, and their deficiency is considered a modifiable risk factor for depression [7]. The levels of PUFAs in the body are largely dependent on dietary intake. Over the past century, there has been a sharp increase in the ω6/ω3 PUFA ratio in the diet, which is believed to be associated with the increasing prevalence of depression [[8], [9], [10]]. Low ω3 PUFA levels and high ω6/ω3 PUFA ratios have been observed in adolescents with depression, and may contribute to an imbalanced inflammatory state [[11], [12], [13]] with potential causal implications for depression [14]. Furthermore, although several randomized controlled trials (RCTs) examining ω3 PUFA supplementation in children and adolescents with depression have demonstrated heterogeneity, they nonetheless indicate promising therapeutic effects [[15], [16], [17], [18], [19], [20], [21], [22], [23]]. In our recent study, in accordance with the clinical practice guidelines established by the International Society for Nutritional Psychiatry Research (ISNPR) [24], we found that adjuvant ω3 PUFA therapy significantly enhanced the effects of antidepressants, leading to marked improvements in depressive symptoms and cognitive function in depressed adolescents [23].

A few studies have preliminarily investigated the mechanisms by which ω3 PUFAs may contribute to the treatment of adolescent depression. Trebatická and colleagues reported that 12 weeks of ω3 PUFA supplementation significantly influenced lipoprotein, thromboxane B2, brain-derived neurotrophic factor, and 5-hydroxytryptophan levels and alleviated oxidative stress in adolescents with depression or mixed anxiety depression [[25], [26], [27], [28]]. Another research team found that ω3 PUFAs altered emotion-related network transferring efficiency and emotion-generated corticolimbic functional connectivity, but was not superior to placebo for reducing depressive symptoms in depressed adolescents at high risk for bipolar disorder [29,30]. Notably, the samples included in these studies are often confounded by other comorbid conditions, complicating the generalization of findings to the broader adolescents with depression. Moreover, the understanding of the effects of ω3 PUFAs on adolescent depression is still evolving, and the links between the molecular changes induced by ω3 PUFAs and improvements in clinical symptoms are rarely established, making it challenging to identify the biochemical underpinnings underlying their therapeutic effects. Importantly, given the inconsistent clinical outcomes of ω3 PUFA treatment, there is an urgent need to identify a biologically homogeneous subgroup of depressed adolescents who may benefit most from this intervention. It is suggested that inflammation and oxidative stress may serve as promising biomarkers for predicting therapeutic response to ω3 PUFA supplementation in adult patients with depression [31,32], but whether this can be generalized to adolescent patients remains to be investigated.

In this study, we performed untargeted metabolomic analysis to investigate the peripheral metabolic changes (plasma and erythrocyte membranes) in response to ω3 PUFA supplementation in adolescents with depression. Our goal was to identify the key metabolites or metabolic pathways associated with improvements of clinical symptoms. Importantly, we sought to identify a subgroup of patients who may exhibit enhanced responsiveness to ω3 PUFA supplementation. This research aimed to elucidate the molecular mechanisms underlying ω3 PUFA therapy for adolescent depression and to inform personalized treatment strategies.

## 2 Material and methods

### 2.1 Study design and participants

An open-label and non-placebo RCT to determine the efficacy of ω3 PUFA adjuvant therapy in adolescents with depression was conducted, where the study design and clinical outcome have been previously described in detail [23]. This study enrolled adolescents aged 13–24 years with depression according the diagnostic criteria of ICD10 (International Classification of Diseases, 10th Revision) from the Fourth People's Hospital of Wuhu in Anhui Province, China. Briefly, 71 patients were randomly assigned to two groups (ratio 1:1): receiving paroxetine alone (20 mg/d, the Paxil group) or fish oil supplementation (2700 mg/d of ω3 PUFAs, including 1941 mg of EPA and 759 mg of DHA) and equal dose of paroxetine (the ω3 + Paxil group) for 12 weeks. Depression severity was assessed by Montgomery-Asberg Depression Rating Scale (MADRS), and cognitive function was assessed by Montreal Cognitive Assessment (MoCA) and Wechsler Memory Scale (WMS). Venous blood samples from 51 patients were collected after 12-h overnight fast both at baseline and at week 12, followed by plasma and red blood cells isolated for subsequent analysis. This study was carried out in accordance with Declaration of Helsinki and approved by the Medical Ethical Committee of the Fourth People's Hospital of Wuhu (No. 2020-KY-15). The RCT was registered in the ISRCTN registry with ID ISRCTN68038781. All participants provided written informed consent, and for those under 18, consent was given by their parents.

### 2.2 Untargeted metabolomic analysis of plasma

Plasma protein was removed by addition of methanol/acetonitrile (1:1, by vol.), freezing, and centrifugation. Then the supernatant was dried and redissolved in 50% methanol solution for mass spectrometry detection. Untargeted metabolomic analysis was performed by Vanquish UHPLC system & Q Exactive plus Mass spectrometer (Thermo Fisher Scientific, MA, USA) with a HSS T3 column (100 ∗ 2.1 mm, 1.7 μm, Waters, MA, USA) in both positive and negative modes. The mobile phase A was water with 0.1% formic acid (v/v), and mobile phase B was methanol/acetonitrile/isopropanol (2:2:1, by vol.) with 0.1% formic acid (v/v). The elution gradient was 99/1∼0/90 in 12 min and hold in 0/90 for 12–13 min. The column was maintained at 45 °C. The flow rate was 0.4 mL/min. Scan mode of QE/MS was data dependent acquisition mode (1 full scan followed by 5 MS/MS scans) and full scan range was 70–1000 amu. The resolutions of full scan and dd-MS/MS were 70000 and 17500 respectively. Spray voltage was 3.2 kV in positive mode and 2.8 kV in negative mode. Capillary temperature was 320 °C. Data were processed by Progenesis QI software (Waters) for peak picking, alignment, and metabolite identification to produce peak intensities for retention time (RT) and *m*/*z* data pairs.

### 2.3 Lipidomic analysis of erythrocyte membrane

The method for erythrocyte membrane lipidomic was in accord with our study previously published [33]. In short, erythrocyte membrane was prepared by incubating overnight with 2 mL of Tris-HCL (pH = 7.4, 10 mM) to burst, followed by washing three times with PBS. The membrane lipids were then extracted with 300 μL of methanol/chloroform mixtures (1:2, v/v) and dissolved in 100 μL of dichloromethane/isopropanol/methanol mixtures (1:1:2, v/v/v) after drying. Lipidomic analysis was performed by UHPLC QE/MS with a BEH C18 column (100 ∗ 2.1 mm, 1.7 μm, Waters) in both positive and negative modes. Data were processed by Lipidsearch 4.2 (Thermo Fisher Scientific), and peak intensities for RT and *m*/*z* data pairs were obtained. The cell membrane lipids were quantified by six lipid standards, including sphingomyelin (SM, d18:1/12:0), ceramide (Cer, d18:1/18:0), phosphatidylserine (PS, 17:0/17:0), phosphatidyl ethanolamine (PE, 17:0/17:0), phosphatidylcholine (PC, 19:0/19:0) and triglyceride (TG, 17:0/17:1/17:0) D5.

### 2.4 Statistical analysis

Principal component analysis (PCA) and orthogonal partial least square discriminant analysis (OPLS-DA) were conducted for multivariate statistical analysis. The plasma metabolites or membrane lipids with variable importance in the projection (VIP) > 1 were thought to contribute significantly to the difference. Paired Wilcox test was used for comparison before and after treatment, and Mann-Whitney test was used for comparison between groups. The p-values were corrected using false discovery rate (FDR) method. Differential metabolites and lipids were identified using multivariate and univariate statistical significance criteria (VIP>1 and FDR<0.05). Enrichment analysis of differential metabolites and lipids was performed by hypergeometric distribution. Pathway analysis was performed using the online website MetaboAnalyst 5.0. Spearman's correlation coefficient was applied to evaluate the relationships between metabolites and clinical symptoms. A Cox regression model was employed to calculate the hazard ratio (HR) with a 95% confidence interval (CI) for analyses of clinical response, grouping based on the direction of metabolite changes, with the group showing reduced metabolite levels serving as the reference. Linear mixed-effects modeling was used to compare trends in MADRS, MoCA, and WMS scores over time between the groups, with adjusting for sex, age, body mass index (BMI), first-episode status, history of suicide, and history of non-suicidal self-injury. A p-value of less than 0.05 (or FDR less than 0.05) was considered statistically significant. R 4.3.1 were used for all statistical analysis.

## 3 Results

### 3.1 Demographic and clinical characteristics

Seventy-one patients were recruited and randomized to the ω3 + Paxil or Paxil groups to evaluate the efficacy of ω3 PUFAs in the treatment of adolescent depression [23]. Venous blood samples were collected from 51 participants for further molecular investigation in this study, the demographic and clinical information of which were not significantly biased compared with those of 71 patients (Table 1). Age, sex, BMI, first-episode status, and history of suicide and non-suicidal self-injury were not significantly different between the ω3 + Paxil (n = 27) and Paxil (n = 24) groups. In terms of clinical symptoms, no significant differences were observed in the MADRS, MoCA, or WMS scores between the two groups at baseline. After 12 weeks, the ω3 + Paxil group presented significantly lower MADRS scores and significantly higher clinical response rate (reduction in MADRS scores ≥50%), MoCA scores, and WMS scores compared with the Paxil group (Table 1), indicating that the adolescents who received ω3 PUFA supplementation exhibited more alleviation of depressive symptoms and better improvement of cognitive function than those who did not receive ω3 PUFA supplementation.

**Table 1:** Demographics and clinical characteristics.

Characteristics	All samples (N = 71)	This study (N = 51)	*p*	Paxil (N = 24)	ω3 + Paxil (N = 27)	*p*
Sex (Male/Female)^a^	29/42	23/28	0.777	7/17	16/11	0.061
Age (years, median, IQR)^b^	17.0 (3.5)	17.0 (2.5)	0.706	16.5 (3.3)	17.0 (2.5)	0.640
BMI (kg/m^2^, median, IQR)^b^	20.8 (4.7)	21.0 (3.9)	0.665	21.1 (5.5)	20.9 (3.8)	0.610
First episode (n, %)^a^	54 (76.1%)	41 (80.4%)	0.728	18 (75.0%)	23 (82.1%)	0.575
History of suicide (n, %)^a^	17 (23.9%)	11 (21.6%)	0.929	6 (25.0%)	5 (17.9%)	0.825
History of non-suicidal self-injury (n, %)^a^	45 (63.4%)	31 (60.8%)	0.918	16 (66.7%)	15 (53.6%)	0.600
MADRS scores at baseline^c^	25.9 ± 4.8	25.7 ± 5.0	0.811	25.9 ± 5.4	25.5 ± 4.7	0.760
MADRS scores at week 12^c^	15.2 ± 7.1	14.9 ± 7.5	0.838	17.8 ± 7.9	12.4 ± 6.2	**0.009**
Response at week 12 (n, %)^a^	28 (39.4%)	21 (41.2%)	0.995	5 (20.8%)	16 (59.3%)	**0.012**
Remission at week 12 (n, %)^a^	20 (28.2%)	15 (29.4%)	1	5 (20.8%)	10 (37.0%)	0.337

MoCA scores at baseline^b^	28.0 (3.0)	28.0 (3.0)	0.786	28.0 (3.0)	28.0 (2.0)	0.625
MoCA scores at week 12^b^	30.0 (1.0)	30.0 (1.0)	0.671	29.0 (2.0)	30.0 (0.0)	**<0.001**
WMS scores at baseline^c^	97.3 ± 18.9	98.4 ± 19.9	0.760	98.5 ± 21.7	98.3 ± 18.7	0.966
WMS scores at week 12^c^	114.1 ± 13.6	113.4 ± 14.9	0.784	108.2 ± 14.7	118.0 ± 13.8	**0.017**

Continuous variables are presented as mean ± standard deviation (SD) or median (interquartile range, IQR). The numbers in the bold are statistically significant. ω3, Omega-3 Polyunsaturated Fatty Acids; BMI, Body Mass Index; MADRS, Montgomery-Asberg Depression Rating Scale; MoCA, Montreal Cognitive Assessment; WMS, Wechsler Memory Scale. ^a^ Chi-Square test, ^b^ Mann-Whitney *U* test, ^c^ T test.

### 3.2 Adjuvant ω3 PUFA supplementation significantly affected the plasma metabolomic profile

**Fig. 1:**
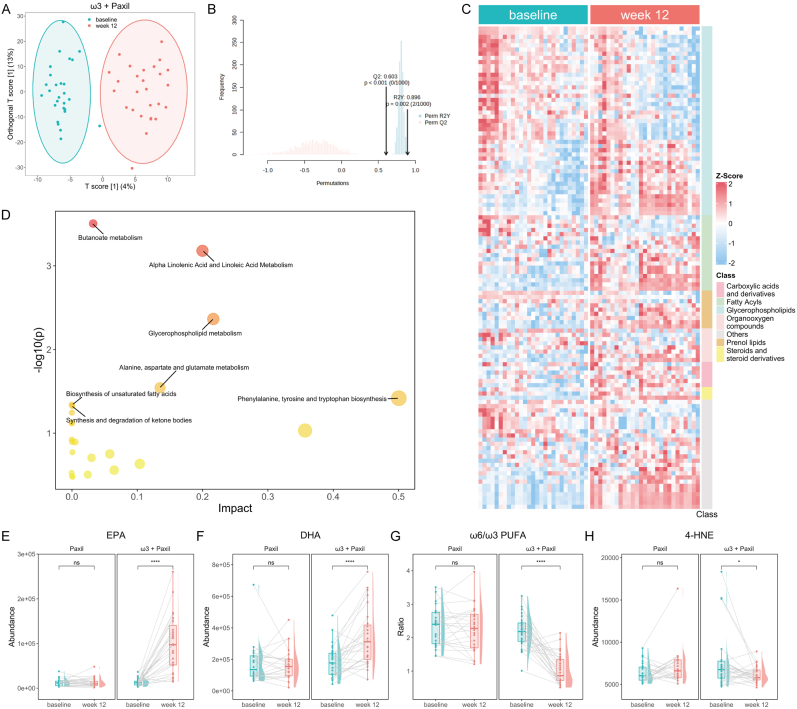
**Adjuvant ω3 PUFA supplementation significantly affected the plasma metabolomic profiles, especially lipids metabolism.** (A) OPLS-DA scores plot of plasma metabolomic between baseline and week 12 in ω3 + Paxil group. (B) Permutation testing of the OPLS-DA model. (C) Heatmap and (D) pathway analysis of the differential metabolites between baseline and week 12 in ω3 + Paxil group. The size and color of each circle are based on pathway impact value and p-value, respectively. The changes of (E) EPA levels, (F) DHA levels, (H) ω6/ω3 PUFA balance, and (I) 4-HNE levels from baseline to week 12 in ω3 + Paxil and Paxil groups, respectively. OPLS-DA, Orthogonal Partial Least Squares-Discriminant Analysis; EPA, Eicosapentaenoic Acid; DHA, Docosahexaenoic Acid; PUFA, Polyunsaturated Fatty Acids; 4-HNE, 4-Hydroxynonenal. ∗p < 0.05, ∗∗∗∗p < 0.0001.

We conducted an untargeted metabolomic analysis of plasma samples from depressed adolescents at baseline and after 12 weeks of treatment, identifying a total of 1182 metabolites. PCA (Fig. S1A) and OPLS-DA (R2Y: 0.896, Q2: 0.603, Fig. 1A and B) of the 1182 metabolites revealed a distinct difference in the plasma metabolome between baseline and week 12 in the ω3 + Paxil group, with 115 metabolites showing significant alterations (Table S1). In contrast, the PCA model revealed no significant separation (Fig. S1B) before and after treatment in the Paxil group, and no metabolites exhibited significant changes with FDRs greater than 0.05, indicating that paroxetine alone did not significantly impact the plasma metabolome. Furthermore, comparison of the plasma metabolome between the ω3 + Paxil and Paxil groups revealed no notable differences at baseline (Fig. S1C), but a trend toward differentiation was observed at week 12 (Fig. S1D). These results suggest that the alterations in the plasma metabolome in the ω3 + Paxil group were primarily attributable to the effect of ω3 PUFAs.

The heatmap was used to illustrate the overall profile of the differentially expressed metabolites after ω3 PUFA supplementation, with glycerophospholipids and fatty acids being the most altered categories (Fig. 1C). Enrichment analysis provided further evidence that the differential metabolites were significantly enriched in "lipids and lipid-like molecules", especially "glycerophospholipids" (Fig. S2A). Moreover, pathway analysis identified several lipid metabolism pathways that were significantly altered, including "glycerophospholipid metabolism", "alpha-linolenic acid and linoleic acid metabolism", and "biosynthesis of unsaturated fatty acids" (Fig. 1D). There was a significant increase in the levels of free EPA and DHA (Fig. 1E and F) with a concomitant decrease in the ω6/ω3 PUFA ratio (Fig. 1G). Similarly, the incorporation of EPA and DHA into glycerophospholipids was increased, potentially replacing ω6 PUFAs, especially arachidonic acid (C20:4, AA) (Fig. S2B), which affects the levels of pro-inflammatory and anti-inflammatory metabolites derived from these PUFAs. The levels of leukotriene B5 (LTB5), a downstream metabolite of EPA with anti-inflammatory properties, significantly increased after ω3 PUFA treatment (Fig. S2C). In addition, given the significant antioxidant properties of ω3 PUFAs, we observed significant decreases in the levels of 4-hydroxynonenal (4-HNE, Fig. 1H) and *trans*-4,5-epoxy-2(E)-decenal (Fig. S2D), both of which are products of lipid peroxidation. Notably, after 12 weeks of treatment, the plasma level of serotonin in the ω3 + Paxil group was significantly greater than that in the Paxil group (Fig. S3), which may account for the enhanced antidepressant effect of ω3 PUFAs. Collectively, these results suggest that lipid metabolism was significantly affected after ω3 PUFA supplementation in depressed adolescents.

### 3.3 Adjuvant ω3 PUFA supplementation affected glycerophospholipid metabolism in the cell membrane

**Fig. 2:**
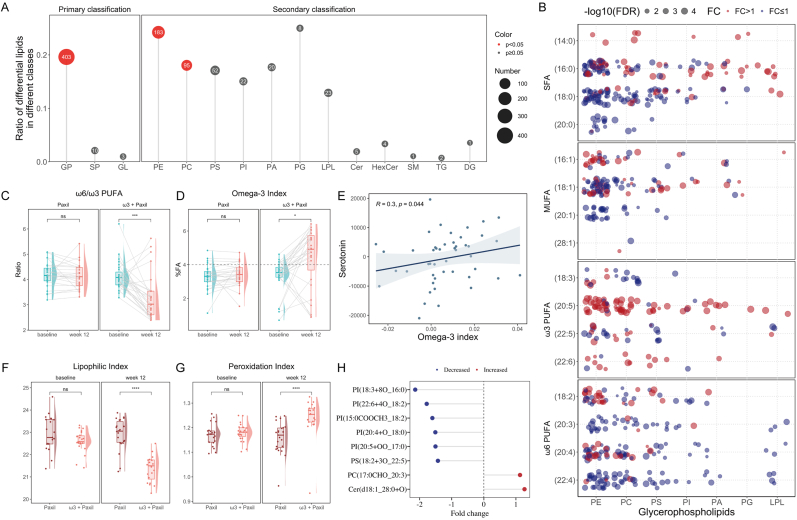
**Adjuvant ω3 PUFA supplementation affected cell membrane glycerophospholipid composition, improved ω6/ω3 PUFA balance, and enhanced membrane function.** (A) Enrichment analysis of differential lipids between baseline and week 12 in ω3 + Paxil group. (B) Characteristics of differential glycerophospholipids after adjuvant ω3 supplementation. The changes of (C) ω6/ω3 PUFA and (D) omega-3 index from baseline to week 12 in ω3 + Paxil and Paxil groups, respectively. (E) The association between the increase of serotonin levels and the increase of omega-3 index. The differences of (F) lipophilic index and (G) peroxidation index between ω3 + Paxil and Paxil groups at baseline and week 12, respectively. (H) Significant changes in oxidized lipids of erythrocyte membrane after adjuvant ω3 PUFA supplementation. GP, Glycerophospholipid; SP, Sphingolipid; GL, Glycerolipid; PE, Phosphatidylethanolamine; PC, Phosphatidylcholine; PS, Phosphatidylserine; PI, Phosphatidylinositol; PA, Phosphatidic Acid; PG, Phosphatidylglycerol; LPL, Lysophospholipid; Cer, Ceramide; HexCer, Hexosyl Ceramide; SM, Sphingomyelin; TG, Triglyceride; DG, Diglyceride; SFA, Saturated Fatty Acids; MUFA, Monounsaturated Fatty Acids; PUFA, Polyunsaturated Fatty Acids; FC, Fold Change; FDR, False Discovery Rate. ∗p < 0.05, ∗∗∗p < 0.001, ∗∗∗∗p < 0.0001.

Given that the substantial alterations in plasma glycerophospholipids and reduced lipid peroxidation are typically derived from membrane lipids, we conducted a further investigation of the lipid composition of cell membranes. A total of 2837 membrane lipids were detected and quantified, with PE, PC, and PS comprising the top three in terms of both the number and abundance of individual species (Fig. S4). OPLS-DA revealed significant changes in the membrane lipid profile after 12 weeks in the ω3 + Paxil group (R2Y: 0.866, Q2: 0.616) but not in the Paxil group (Fig. S5). A total of 416 membrane lipids were significantly altered in the ω3 + Paxil group (Table S2), whereas the FDRs of all lipids in the Paxil group were greater than 0.05 (Fig. S6). Subsequent enrichment analysis revealed a significant enrichment of glycerophospholipids among the differential lipids (Fig. 2A), of which PE and PC accounted for 43.99% and 22.84%, respectively. Among the glycerophospholipids that exhibited significant changes in the ω3 + Paxil group, we observed pervasive increases in the incorporation of EPA and DHA, accompanied by a decrease in ω6 PUFAs (Fig. 2B). Therefore, we delved deeper into the changes in membrane lipids from the perspective of fatty acid chain composition.

In terms of fatty acids (% of total fatty acids), the levels of three were increased (EPA, C18:3, and C16:1), whereas the levels of five were decreased (AA, C22:4, C18:0, C18:1, and C20:1) after 12 weeks of ω3 PUFA supplementation (Table 2). Although the increase in DHA level in the ω3 + Paxil group was not statistically significant (p = 0.062), the change from baseline to week 12 (from 2.76% to 3.41%) was significantly greater than that in the Paxil group (from 2.52% to 2.62%). In the Paxil group, only C16:1 level increased significantly, with no notable difference in the increase between the Paxil and ω3 + Paxil groups. A significant increase in overall ω3 PUFA levels and a decrease in total ω6 PUFA levels in the membrane were observed after 12 weeks of ω3 PUFA supplementation, accompanied by a significant decrease in the ω6/ω3 PUFA ratio (Fig. 2C). Notably, the omega-3 index, which refers to the combination of membrane EPA and DHA levels, is considered associated with depression risk when it is less than 4% [34]. At baseline, 81.5% (22 out of 27) of patients in the ω3 + Paxil group had an omega-3 index below 4%. We observed a significant increase in this index from baseline to week 12, with the mean rising from 3.3% to 4.4% (Fig. 2D). By week 12, only 25.9% (7 out of 27) remained below 4%. In addition, we found that an increase in the omega-3 index was significantly positively correlated with an increase in the plasma serotonin level (Fig. 2E).

**Table 2:** Changes of fatty acids in erythrocyte membrane pre and post supplementation with or without ω3 PUFAs in adolescent depression.

Fatty acid	Paxil			ω3 + Paxil			Change from baseline		
	baseline	week12	*p*	baseline	week12	*p*	Paxil	ω3 + Paxil	*p*
SFA	39.29 (1.08)	39.18 (0.79)	0.178	39.01 (0.71)	38.88 (3.50)	0.530	−0.27 (0.96)	−0.26 (1.13)	0.687
(14:0)	0.90 (0.17)	0.88 (0.09)	0.900	0.91 (0.20)	0.88 (0.96)	0.934	−0.02 (0.17)	0 (0.23)	0.874
(16:0)	16.19 (0.82)	16.14 (1.11)	0.855	16.12 (1.06)	16.29 (2.22)	0.279	−0.10 (1.14)	0.28 (1.49)	0.404
(18:0)	17.78 (0.99)	17.23 (1.26)	0.290	17.16 (1.04)	16.81 (1.20)	**0.044**	0 (1.33)	−0.52 (1.31)	0.543
(20:0)	0.65 (0.18)	0.62 (0.09)	0.790	0.62 (0.12)	0.68 (0.18)	0.141	0.01 (0.16)	0.06 (0.10)	0.185
MUFA	26.14 (0.47)	26.21 (0.51)	0.390	26.58 (0.80)	26.22 (1.71)	0.135	0.06 (0.63)	−0.42 (1.03)	**0.026**
(16:1)	3.12 (0.46)	3.30 (0.49)	**0.002**	3.21 (0.28)	3.43 (0.56)	**0.036**	0.12 (0.34)	0.20 (0.66)	0.933
(18:1)	18.95 (0.64)	18.91 (0.71)	0.584	19.17 (0.81)	18.74 (0.62)	**5.36E-04**	−0.09 (0.74)	−0.48 (0.81)	**0.015**
(20:1)	1.15 (0.19)	1.12 (0.23)	0.922	1.13 (0.16)	1.01 (0.20)	**0.007**	0 (0.12)	−0.12 (0.21)	**0.018**
(28:1)	0.87 (0.13)	0.89 (0.28)	1.000	0.86 (0.12)	0.97 (0.45)	0.500	0 (0.22)	0.01 (0.37)	0.581

ω3 PUFA	6.03 (0.87)	6.13 (0.95)	0.317	6.16 (0.74)	7.87 (3.57)	**0.016**	0.11 (0.49)	1.96 (1.66)	**5.30E-04**
(18:3)	1.22 (0.11)	1.22 (0.25)	0.726	1.22 (0.10)	1.27 (0.14)	**0.039**	−0.02 (0.16)	0.03 (0.13)	0.111
(20:5)	0.88 (0.17)	0.84 (0.29)	0.663	0.86 (0.22)	1.61 (1.23)	**4.75E-05**	−0.03 (0.16)	0.77 (1.05)	**4.71E-05**
(22:5)	1.29 (0.18)	1.30 (0.14)	0.491	1.29 (0.22)	1.50 (0.61)	0.058	−0.02 (0.15)	0.20 (0.35)	**0.008**
(22:6)	2.52 (0.66)	2.62 (0.73)	0.084	2.76 (0.64)	3.41 (1.41)	0.062	0.11 (0.33)	0.52 (0.65)	**0.007**
ω6 PUFA	24.64 (1.64)	24.74 (1.13)	0.855	24.64 (1.05)	22.99 (3.49)	**0.005**	0.02 (0.81)	−1.34 (1.91)	**0.002**
(18:2)	7.08 (1.03)	7.20 (0.85)	0.060	7.08 (1.15)	7.30 (1.11)	0.052	0.17 (0.72)	0.16 (0.39)	0.801
(20:3)	2.64 (0.20)	2.62 (0.30)	0.623	2.58 (0.42)	2.69 (0.36)	0.290	0 (0.25)	0.04 (0.24)	0.211
(20:4)	11.93 (1.08)	11.66 (0.71)	0.473	11.71 (1.13)	10.72 (2.82)	**0.006**	−0.04 (0.68)	−0.95 (1.36)	**0.007**
(22:4)	3.15 (0.51)	3.13 (0.40)	0.768	3.04 (0.51)	2.30 (0.68)	**2.52E-06**	0.01 (0.26)	−0.69 (0.57)	**8.41E-06**

Fatty acid composition (% of total fatty acids) is presented as median (interquartile range). The numbers in the bold are statistically significant. SFA, Saturated Fatty Acids; MUFA, Monounsaturated Fatty Acids; PUFA, Polyunsaturated Fatty Acids.

Importantly, changes in the composition of membrane lipids affect the function of the cell membrane. The lipophilic index, a comprehensive index of membrane fluidity, was lower in the ω3 + Paxil group than in the Paxil group at week 12 (Fig. 2F), implying that ω3 PUFAs facilitated improved membrane fluidity. Moreover, the peroxidation index at week 12 was significantly greater in the ω3 + Paxil group than in the Paxil group (Fig. 2G), indicating that ω3 PUFAs increased the membrane antioxidant capacity. Notably, it is observed that several oxidized lipids decreased significantly in the ω3 + Paxil group after treatment (Fig. 2H), suggesting that ω3 PUFAs alleviated membrane oxidative stress and may account for the decrease of 4-HNE in plasma. Collectively, these results suggest that ω3 PUFA supplementation significantly increased the membrane ω3 PUFA composition and improved membrane function, which may underlie the antidepressant efficacy observed with ω3 PUFA treatment.

### 3.4 Changes in lipid metabolism and cell membrane function were associated with improved clinical symptoms

**Fig. 3:**
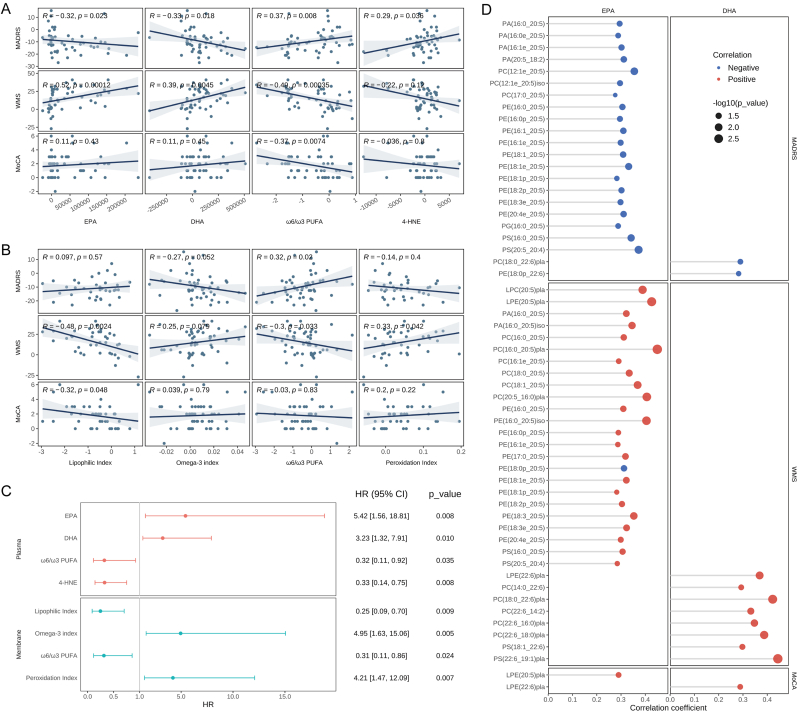
**Associations between changes of clinical outcomes and changes of lipid indicators from baseline to week 12.** (A) Associations between changes of clinical outcomes and changes of EPA levels, DHA levels, ω6/ω3 PUFA ratio, and 4-HNE levels in Plasma. (B) Associations between changes of clinical outcomes and changes of lipophilic index, omega-3 index, ω6/ω3 PUFA ratio, and peroxidation index in cell membrane. (C) Associations between changes of lipid indicators and clinical response. (D) Significant associations between changes of clinical outcomes and changes of phospholipids levels containing EPA or DHA. The "pla" label indicates phospholipids in plasma, while the "iso" label refers to isomers on cell membrane phospholipids. MADRS, Montgomery-Asberg Depression Rating Scale; MoCA, Montreal Cognitive Assessment; WMS, Wechsler Memory Scale; EPA, Eicosapntemacnioc Acid; DHA, Docosahexaenoic Acid; PUFA, Polyunsaturated Fatty Acids; 4-HNE, 4-Hydroxynonenal; HR, Hazard Ratios; CI, Confidence Interval; PA, Phosphatidic Acid; PC, Phosphatidylcholine; PE, Phosphatidylethanolamine; PG, Phosphatidylglycerol; PS, Phosphatidylserine; LPC, Lysophosphatidylcholine; LPE, Lysophosphatidylethanolamine.

To investigate whether the changes in lipids induced by adjuvant ω3 PUFA supplementation account for the improvements in clinical symptoms, we conducted correlation analyses between the changes in lipid indicators and changes in clinical symptom scale scores from baseline to week 12. The results revealed a significant correlation between changes in plasma EPA and DHA levels and changes in the MADRS and WMS scores (Fig. 3A). Meanwhile, changes in the MADRS, MoCA, and WMS scores were significantly correlated with changes in the plasma ω6/ω3 PUFA ratio (Fig. 3A). Similarly, notable correlations were observed between changes in the MADRS and WMS scores and those in the membrane ω6/ω3 PUFA ratio (Fig. 3B). Moreover, changes in the plasma 4-HNE levels were significantly positively correlated with changes in the MADRS score (Fig. 3A), and changes in the membrane peroxidation index were significantly positively correlated with changes in the WMS score (Fig. 3B). Lipophilic index changes were significantly negatively correlated with changes in the MoCA and WMS scores (Fig. 3B). We further found that the increases in ω3 PUFA content and peroxidation index and the decreases in ω6/ω3 PUFA ratio, 4-HNE levels, and lipophilic index contributed to higher clinical response (Fig. 3C).

Furthermore, we observed that alterations in phospholipids containing EPA or DHA were significantly negatively correlated with changes in MADRS scores and positively correlated with changes in MoCA and WMS scores (Fig. 3D). Notably, phospholipids containing EPA demonstrated widespread correlations with clinical symptom improvements, whereas those containing DHA showed a comparatively limited association. Additionally, results from the Cox regression model indicated that patients with increased levels of phospholipids containing EPA were more likely to exhibit a clinical response (Fig. S7). These findings suggest that EPA is more likely the primary component responsible for the antidepressant effects. Therefore, the changes in phospholipid metabolism induced by ω3 PUFAs (particularly EPA), which resulted in the enhancement in antioxidant and anti-inflammatory capacity and membrane fluidity, may contribute to improvements in depressive symptoms and cognitive function in adolescents with depression.

### 3.5 Depressed adolescents with severe lipid oxidation would benefit from ω3 PUFA supplementation

**Fig. 4:**
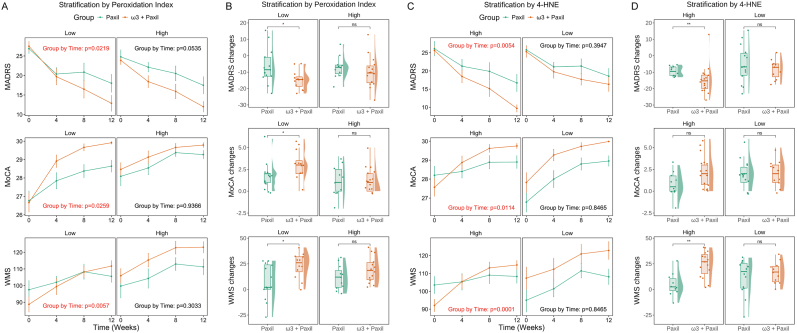
**Depressed adolescents with higher oxidative stress at baseline were more likely to benefit from adjuvant ω3 PUFA supplementation.** (A, B) Comparison of efficacy between ω3 + Paxil and Paxil groups after stratification by peroxidation index. (C, D) Comparison of efficacy between ω3 + Paxil and Paxil groups after stratification by 4-HNE levels. MADRS, Montgomery-Asberg Depression Rating Scale; MoCA, Montreal Cognitive Assessment; WMS, Wechsler Memory Scale; PUFA, Polyunsaturated Fatty Acids; 4-HNE, 4-Hydroxynonenal. ∗p < 0.05, ∗∗p < 0.01.

Given the inconsistent results of ω3 PUFA treatment for adolescent depression, we tried to explored a common molecular basis to identify a subgroup of patients which may benefit from the adjuvant therapy of ω3 PUFA supplementation. We conducted a stratified analysis, dividing the 51 patients into high- and low-level subgroups on the basis of the median level of a metabolite or a combined index at baseline. The baseline peroxidation index was found to predict the treatment response to ω3 PUFA supplementation. Specifically, the interaction term between group and time was statistically significant exclusively in the subgroup with a low baseline peroxidation index, which indicates that the ω3 + Paxil group demonstrated a greater magnitude of change in clinical symptoms compared to the Paxil group (Fig. 4A). Within this subgroup, patients receiving ω3 PUFA supplementation exhibited significantly greater baseline-to-endpoint improvements in MADRS, MoCA, and WMS scores than those receiving paroxetine monotherapy (Fig. 4B). In the subgroup with a high peroxidation index, there were no significant differences in the changes in clinical symptoms between the ω3 + Paxil and the Paxil groups. Similarly, among patients with high baseline 4-HNE levels, those in the ω3 + Paxil group experienced significantly greater improvements in depressive symptoms and cognitive function than those in the Paxil group (Fig. 4C and D). Conversely, in the subset of patients with low baseline 4-HNE levels, adjuvant treatment with ω3 PUFAs did not enhance antidepressant efficacy.

Furthermore, we specifically evaluated oxidized lipids containing EPA or DHA. The patients with higher oxidized EPA levels at baseline showed poorer cognitive functions (Fig. S8A). After the 12-week intervention, the ω3 + Paxil group demonstrated greater increases in the MoCA and WMS scores compared to the Paxil group in subgroup with high oxidized EPA levels (Fig. S8B). In contrast, there were no similar findings when stratified according to oxidized DHA levels (Figs. S8A and S8C). These findings suggest that ω3 PUFA supplementation is more advisable for depressed adolescents with higher levels of lipid oxidation and further support the antidepressant efficacy of EPA rather than 10.13039/100009898DHA.

## 4 Discussion

**Fig. 5:**
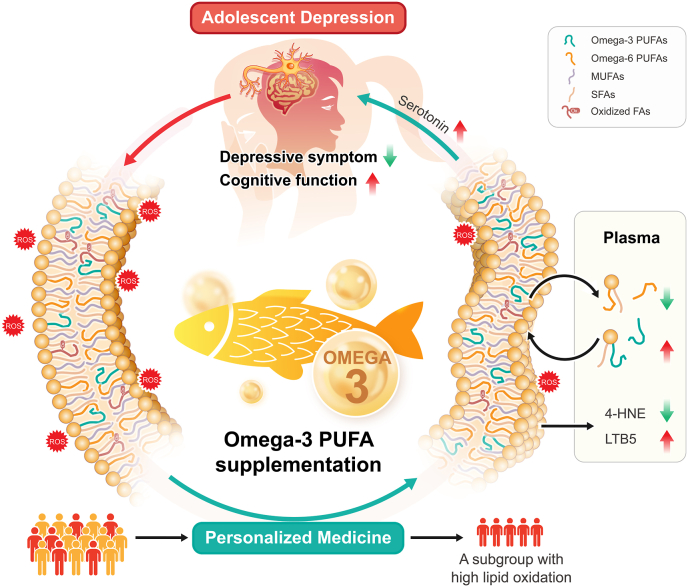
**Schematic of molecular mechanism and biomarker of antidepressant response to ω3 PUFA supplementation in adolescents with depression.** Adolescents with depression exhibit elevated oxidative stress and impaired cell membrane lipids [33]. Supplementation with fish oil to address ω3 PUFA deficiencies in patients improves depressive symptoms and cognitive function. The mechanism involves the restoration of membrane lipid homeostasis, including reduced lipid oxidation and increased membrane fluidity. Additionally, an increased ω6/ω3 PUFA ratio enhances the levels of serotonin and anti-inflammatory metabolites like LTB5, potentially promoting antidepressant effects. Notably, a subgroup of adolescents with high lipid oxidation levels benefits more from ω3 PUFA supplementation. SFA, Saturated Fatty Acids; MUFA, Monounsaturated Fatty Acids; PUFA, Polyunsaturated Fatty Acids; 4-HNE, 4-Hydroxynonenal; LTB5, Leukotriene B5; ROS, Reactive Oxygen Species.

This study investigated the metabolic alterations in adolescents with depression following supplementation with ω3 PUFAs (Fig. 5). Our findings indicate that lipid metabolism was predominantly influenced by ω3 PUFAs, resulting in reduced lipid peroxidation and enhanced membrane fluidity. The changes in lipids, particularly those containing EPA, were closely associated with improvements in depressive symptoms and cognitive function. Importantly, we identified a subgroup of depressed adolescents who responded to ω3 PUFA adjuvant therapy on the basis of lipid oxidation levels.

It is reported that an omega-3 index ≤4% is associated with an elevated risk of depression [34,35]. Additionally, a lower omega-3 index also puts depressed adolescents at greater risk for suicide and cardiovascular disease, the two leading causes of premature death in patients with mood disorders [36,37]. The mean omega-3 index of 3.3% observed in this study was in line with findings from previous research in depressed adolescents [20,21]. Following 12 weeks of ω3 PUFA supplementation, the omega-3 index in adolescents with depression (4.4%) is comparable to that in healthy adolescents (4.2%) [20,29], suggesting that a daily intake of fish oil containing 2.7 g ω3 PUFAs is sufficient to correct the low EPA + DHA status in this population. Interestingly, the omega-3 index in depressed adolescents was lower than that reported in depressed adults (3.9%), which is consistent with the finding that erythrocyte ω3 PUFA levels tend to increase with age [38,39]. Importantly, adolescence represents a crucial period for brain development [40], and a higher omega-3 index is associated with greater brain volume and enhanced synaptic function [29,41], thus underscoring the necessity for additional ω3 PUFA supplementation among adolescent populations.

The impact of ω3 PUFAs is acknowledged to be diverse and multifaceted, confounding the mechanisms underlying their efficacy in the treatment of adolescent depression. Our untargeted plasma metabolomic and membrane lipidomic analyses revealed a pronounced influence of ω3 PUFAs on phospholipid metabolism in depressed adolescents. The brain is particularly enriched in phospholipids for the maintenance of brain structure and function, with ω3 PUFAs accounting for 10%–20% of the total fatty acids [42]. Previous studies have demonstrated that ω3 PUFAs in phospholipids facilitate their transport to the brain [43] and that administering ω3 PUFAs to rats significantly increases their levels in both peripheral and central nervous system tissues [44,45]. Moreover, evidence suggests that the PUFA content in the erythrocyte membrane reflects PUFA distribution in the brain [46,47]. Consequently, we hypothesize that the alterations observed in erythrocyte membranes, which showed close association with the amelioration of clinical symptoms in depressed adolescents, may also correspond to similar changes occurring within the brain.

ω3 PUFAs may exert antidepressant effects by alleviating lipid damage induced by oxidative stress, which is recognized as one of the pathological mechanisms underlying adolescent depression [48,49]. The findings support that both oxidized lipids in the cell membrane and 4-HNE levels in the plasma were significantly reduced after 12 weeks of ω3 PUFA supplementation, indicating a decrease in membrane lipid peroxidation. In addition, ω3 PUFAs significantly enhanced the peroxidation index of the cell membrane, indicating an increased potential to resist oxidative damage, which was lower in adolescents with depression than in healthy controls [33,50]. Existing literature indicates that ω3 PUFAs can reduce the production of reactive oxygen species (ROS) and upregulate the expression of nuclear factor erythroid 2-related factor 2 (Nrf2), a key transcription factor for antioxidant genes [51,52]. Moreover, the high unsaturation levels of ω3 PUFAs render them more susceptible to oxidative stress, leading to the synthesis of electrophilic derivatives that function as signaling molecules to activate Nrf2 [53,54]. This is further supported by the significant decrease in SFA and MUFA levels following ω3 PUFA supplementation, as their oxidation products are less involved in the formation of electrophiles. On the other hand, the balance of ω6 and ω3 PUFAs plays an important role in the regulation of the inflammatory state and is positively correlated with oxidative stress [26]. As reported by our study and others [27], ω3 PUFAs inhibit AA production, incorporation at the sn-2 position of phospholipids, and metabolism to pro-inflammatory eicosanoid acids. Therefore, the reduction in the ω6/ω3 PUFA ratio could enhance the production of anti-inflammatory derivatives from EPA and DHA, such as LTB5 identified in this study, thereby mitigating inflammation and accompanying oxidative stress.

Importantly, we further found that the combined strategy with ω3 PUFA supplementation enhanced the antidepressant efficacy exclusively in depressed adolescents with higher lipid oxidative damage, which may explain the contradictory results concerning the efficacy of ω3 PUFA therapy reported in previous trials. Given the high heterogeneity of clinical symptoms and pathological mechanisms in the depressed population, the ISNPR suggests that stratifying patients into more homogeneous subgroups is a crucial strategy for enhancing treatment effectiveness [24]. It has been reported that the inverse association between ω3 PUFA levels and depressive symptoms was observed among individuals with elevated oxidative stress biomarkers [31]. Similar to our results, a RCT involving coronary artery disease indicated that patients stratified with higher lipid peroxidation levels at baseline experienced more significant improvements in depressive symptoms following 12 weeks of ω3 PUFA treatment [55]. In addition, depressed adult patients with higher inflammation levels at baseline, which is inseparable from oxidative stress [56], have been found to be more responsive to ω3 PUFAs [32,57]. Overall, our study, along with others, supports oxidative stress as a potential biomarker for personalized therapeutic interventions. Moreover, these results further demonstrate that the primary molecular mechanism through which ω3 PUFAs exert their therapeutic effect on adolescent depression may be related to their capacity to enhance antioxidant defenses and reduce lipid oxidation.

Another potential antidepressant pathway of ω3 PUFAs that we identified may be related to the enhanced serotonin system. Previous studies have shown that a deficit in ω3 PUFAs is associated with impaired serotonergic neurotransmission [58], which impacts cognitive and emotional functions, contributing to the pathophysiology of depression [59]. Our study indicated that ω3 PUFA supplementation promoted an increase in serotonin levels, in conjunction with the previous finding that ω3 PUFAs stimulate the serotonin pathway [28]. Specifically, it has been proposed that EPA increases the release of serotonin from presynaptic neurons by inhibiting prostaglandin E2 (PGE2) production derived from AA [60]. Moreover, ω3 PUFAs are thought to facilitate the availability of serotonin receptors in postsynaptic neurons through increased cell membrane fluidity [61]. Consistent with this, our study found that supplementation with ω3 PUFAs in depressed adolescents increased cell membrane fluidity, which was related to the enhanced cognitive function. Notably, this effect is not restricted to serotonin receptors but extends to dopamine receptors and other neurotransmitter receptors [62]. Therefore, these results suggest that the antidepressant mechanism of ω3 PUFAs may be complementary to that of antidepressants, which helps explain why combination therapy with antidepressants and ω3 PUFAs produces an increased therapeutic response [63].

Additionally, it is noteworthy that the changes in phospholipids containing EPA were more closely associated with improvements in clinical symptoms than those containing DHA. Meta-analyses have demonstrated that supplements with a predominance of EPA (>50% EPA) exhibit efficacy in alleviating depressive symptoms, whereas those primarily composed of DHA have shown no significant pooled effects in this regard [[63], [64], [65], [66], [67]]. Evidence from Mendelian randomization analysis also revealed a stronger effect of EPA than DHA on the etiology of depression [68]. EPA is likely to act as a messenger in the central nervous system because of its rapid metabolism to prostanoids, resolvins, and the endocannabinoid eicosapentaenoyl ethanolamide, which have been detected in the brain [69,70]. Notably, the content of EPA in microglia cells appears to be at least two-fold greater than that of DHA, suggesting a specialized role for EPA in these immune cells [69]. Existing evidence indicates that EPA can reduce the activation of microglia and influence the production of cytokines [71]. We found that patients with higher oxidized EPA levels experienced worse cognitive function and better responses to ω3 PUFAs, whereas oxidized 10.13039/100009898DHA did not yield similar benefits, further supporting the essential role of EPA.

The present study has several limitations that must be acknowledged. The sample size was relatively small and potential confounding lifestyle factors (such as diet and exercise) were not meticulously controlled for. In addition, this study was an open-label non-placebo trial, indicating that the findings should be regarded as preliminary. In particular, our analysis of the relationship between high oxidative stress and the response to ω3 PUFAs was exploratory. It is possible that the subgroup of adolescents with depression characterized by high oxidative stress may also benefit from other antioxidant treatments. Moreover, this study used a simple median split to stratify patients, highlighting the need for clear criteria to define "high oxidative stress" in future research.

## 5 Conclusion

Our findings elucidate the significant effects of ω3 PUFAs on phospholipid metabolism in adolescents with depression, particularly the alleviation of lipid oxidation, which may be involved in the antidepressant effects of ω3 PUFAs. Notably, we propose that a specific subgroup of depressed adolescents exhibiting elevated lipid oxidation may derive greater therapeutic benefits from ω3 PUFA supplementation. This research contributes valuable insights into the underlying molecular mechanisms of ω3 PUFA therapy and underscores its potential for personalized treatment in the management of adolescent depression.

## CRediT authorship contribution statement

**Jinfeng Wang:** Writing – review & editing, Writing – original draft, Visualization, Methodology, Investigation, Formal analysis. **Shuhui Li:** Writing – review & editing, Methodology. **Dandan Wang:** Writing – review & editing. **Yan Gao:** Writing – review & editing. **Qian Wang:** Writing – review & editing. **Tianqi Wang:** Methodology. **Guanghai Wang:** Writing – review & editing. **Daihui Peng:** Writing – review & editing. **Yi Qiao:** Funding acquisition. **Jiansong Zhou:** Funding acquisition. **Lei Feng:** Methodology. **Xiaowen Hu:** Writing – review & editing, Visualization, Methodology. **Chunling Wan:** Writing – review & editing, Supervision, Resources, Project administration, Funding acquisition, Data curation, Conceptualization.

## Funding

This work was supported by the 10.13039/501100001809National Natural Science Foundation of China [grant numbers 82471522]; the STI2030-Major Projects [grant numbers 2021ZD0200800]; the Fundamental Research Funds for the Central Universities [grant numbers 2022GDND06]; the STI2030-Major Projects [grant numbers 2021ZD0200700]; and the Natural Science Foundation of Shanghai [grant numbers 23ZR1433300].

## Declaration of competing interest

The authors declare that they have no known competing financial interests or personal relationships that could have appeared to influence the work reported in this paper.

## Data Availability

Data will be made available on request.

## Abbreviations

4-HNE, 4-Hydroxynonenal; AA, Arachidonic Acid; BMI, Body Mass Index; Cer, Ceramide; CI, Confidence Interval; DG, Diglyceride; DHA, Docosahexaenoic Acid; EPA, Eicosapentaenoic Acid; FC, Fold Change; FDR, False Discovery Rate; GL, Glycerolipid; GP, Glycerophospholipid; HexCer, Hexosyl Ceramide; HR, Hazard Ratio; ICD-10, International Classification of Diseases, Version 10; ISNPR, International Society for Nutritional Psychiatry Research; LPC, Lysophosphatidylcholine; LPE, Lysophosphatidylethanolamine; LPL, Lysophospholipid; LTB5, Leukotriene B5; MADRS, Montgomery-Asberg Depression Rating Scale; MoCA, Montreal Cognitive Assessment; MUFA, Monounsaturated Fatty Acids; Nrf2, Nuclear factor erythroid 2-related factor 2; OPLS-DA, Orthogonal Partial Least Squares-Discriminant Analysis; PA, Phosphatidic Acid; PC, Phosphatidylcholine; PCA, Principal Component Analysis; PE, Phosphatidylethanolamine; PG, Phosphatidylglycerol; PGE2, Prostaglandin E2; PI, Phosphatidylinositol; PS, Phosphatidylserine; PUFA, Polyunsaturated Fatty Acids; RCT, Randomized Controlled Trial; ROS, Reactive Oxygen Species; RT, Retention Time; SFA, Saturated Fatty Acids; SM, Sphingomyelin; SP, Sphingolipid; TG, Triglyceride; VIP, Variable Importance in the Projection; WMS, Wechsler Memory Scale.
