# Supplementary material for: Effects of Omega-3 PUFAs on lipid profiles and antioxidant response in depressed adolescents: A metabolomic and lipidomic study
Source: Redox Biol. 2025 Mar 25;82:103617. doi: 10.1016/j.redox.2025.103617 (PMC11997347; doi:10.1016/j.redox.2025.103617)
Supplement: Multimedia component 1 [file mmc1.docx]

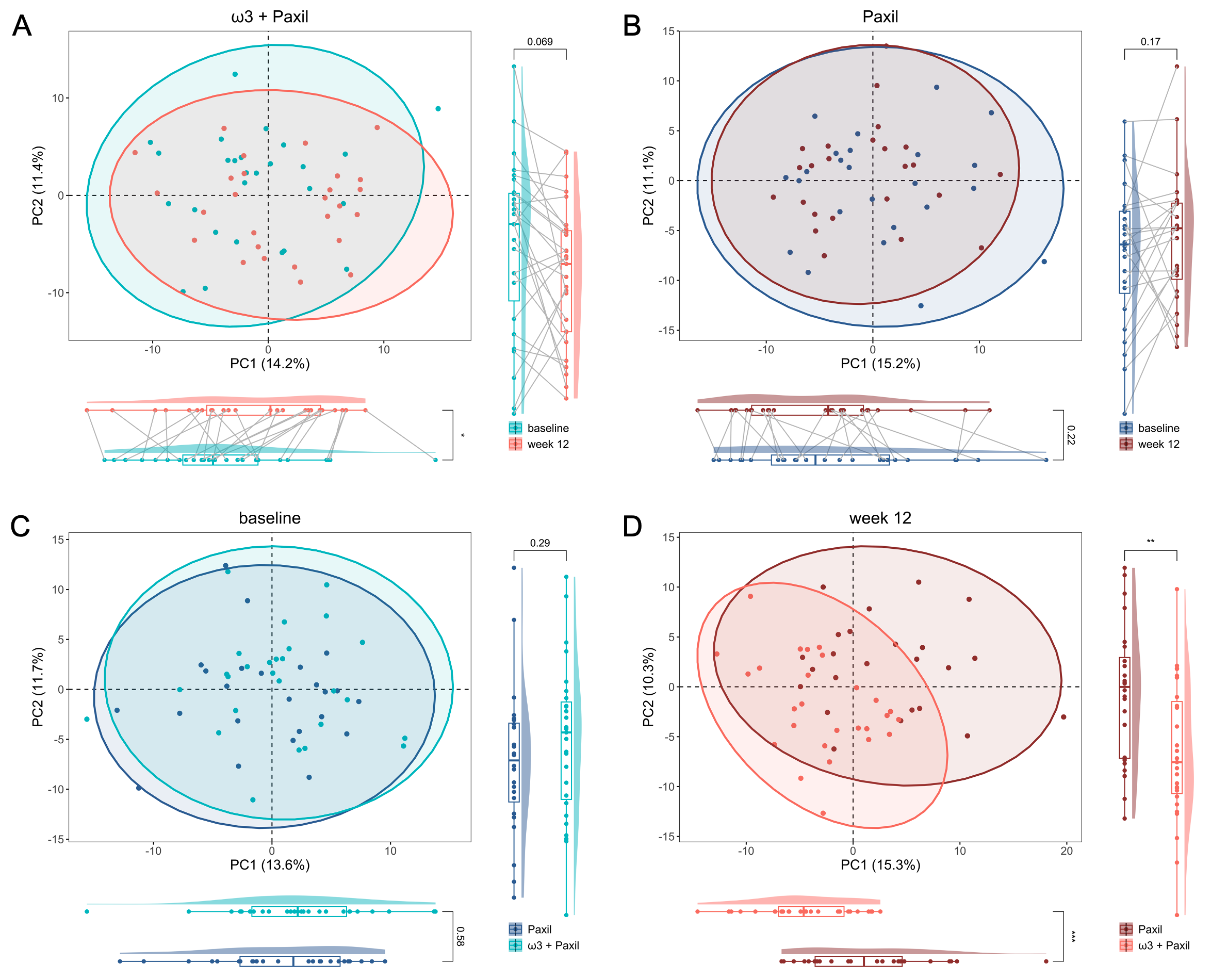


**Figure S1. Plasma metabolomic was obviously different after adjuvant ω3 PUFA supplementation.** PCA score plots of plasma metabolomic and differences of PC1 (bottom) and PC2 (right) (A) between baseline and week 12 in ω3 + Paxil group, (B) between baseline and week 12 in Paxil group, (C) between ω3 + Paxil and Paxil group at baseline, (D) between ω3 + Paxil and Paxil group at week 12. PCA, Principal Component Analysis; PC, Principal Components; ω3 PUFA, Omega-3 Polyunsaturated Fatty Acids. *p<0.05, **p<0.01, ***p<0.001.


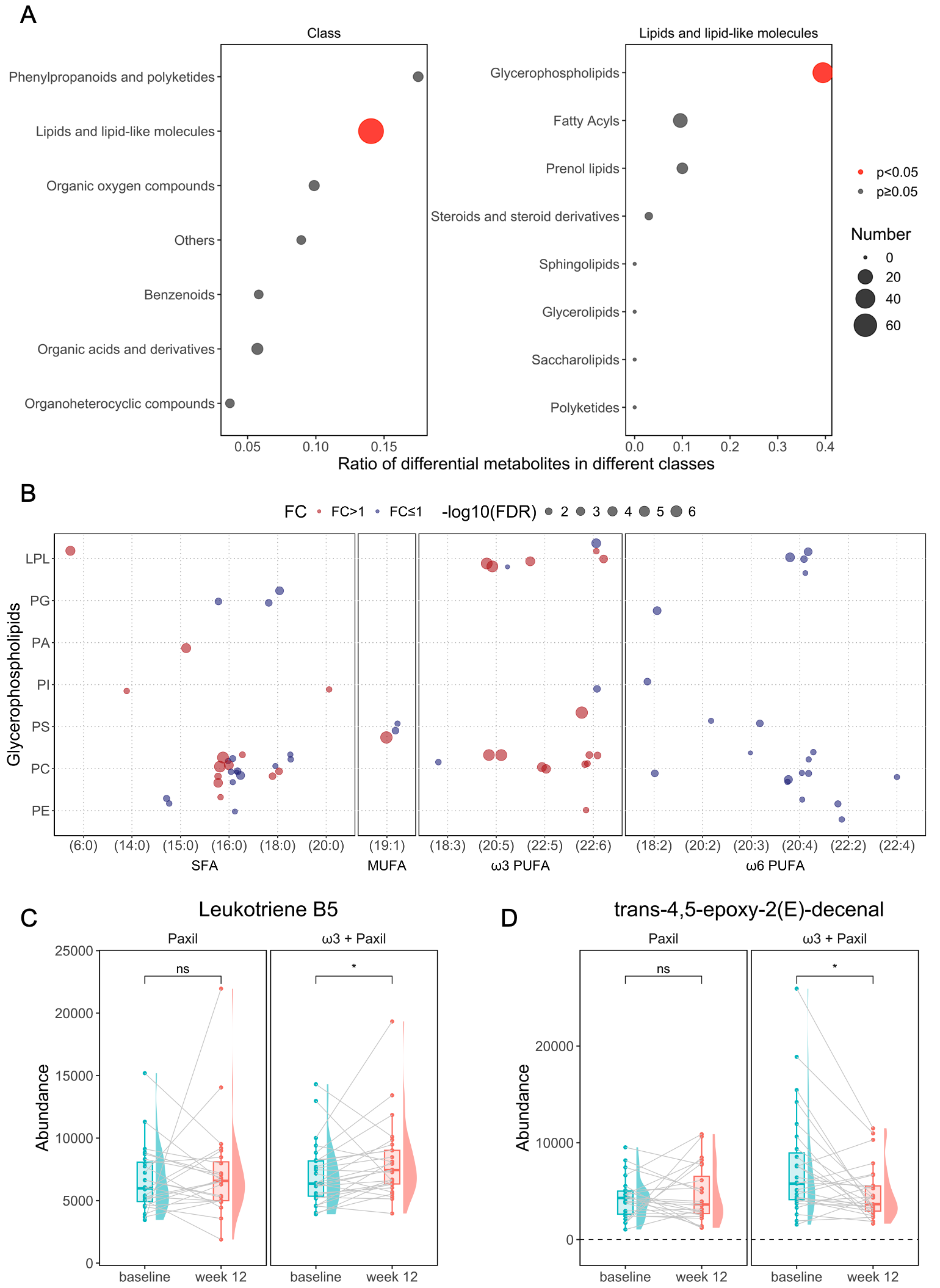


**Figure S2. Adjuvant ω3 PUFA supplementation significantly affected glycerophospholipid metabolism.** (A) Enrichment analysis of the differential metabolites between baseline and week 12 in ω3 + Paxil group. (B) Characteristics of plasma differential glycerophospholipids between baseline and week 12 in ω3 + Paxil group. The changes of (C) leukotriene B5 and (D) trans-4,5-epoxy-2(E)-decenal levels from baseline to week 12 in ω3 + Paxil and Paxil group, respectively. SFA, Saturated Fatty Acids; MUFA, Monounsaturated Fatty Acids; PUFA, Polyunsaturated Fatty Acids; PE, Phosphatidylethanolamine; PC, Phosphatidylcholine; PS, Phosphatidylserine; PI, Phosphatidylinositol; PA, Phosphatidic Acid; PG, Phosphatidylglycerol; LPL, Lysophospholipid; FC, Fold Change; FDR, False Discovery Rate. *p<0.05.


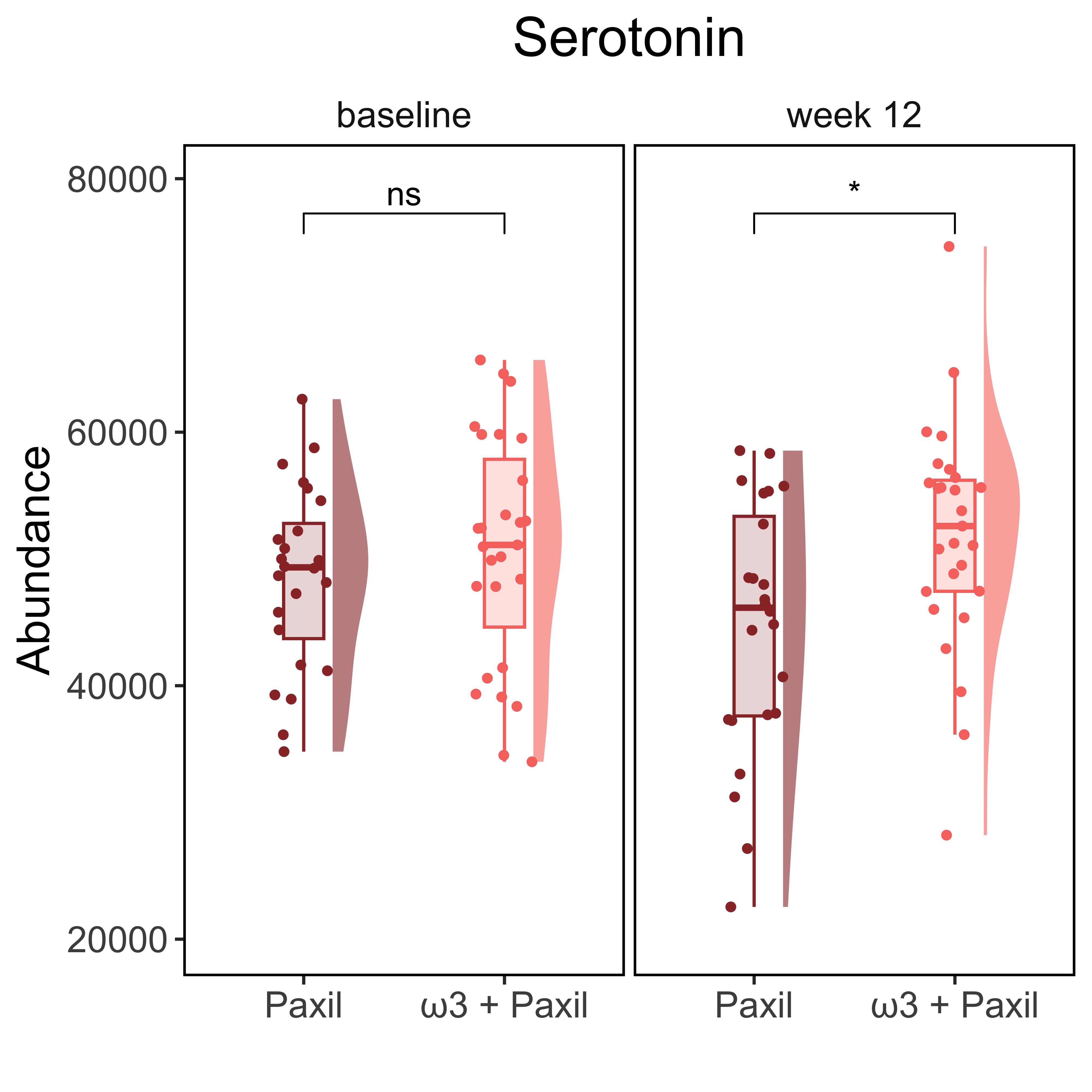


**Figure S3. The differences of serotonin between ω3 + Paxil and Paxil group at baseline and week 12.** *p<0.05.

**
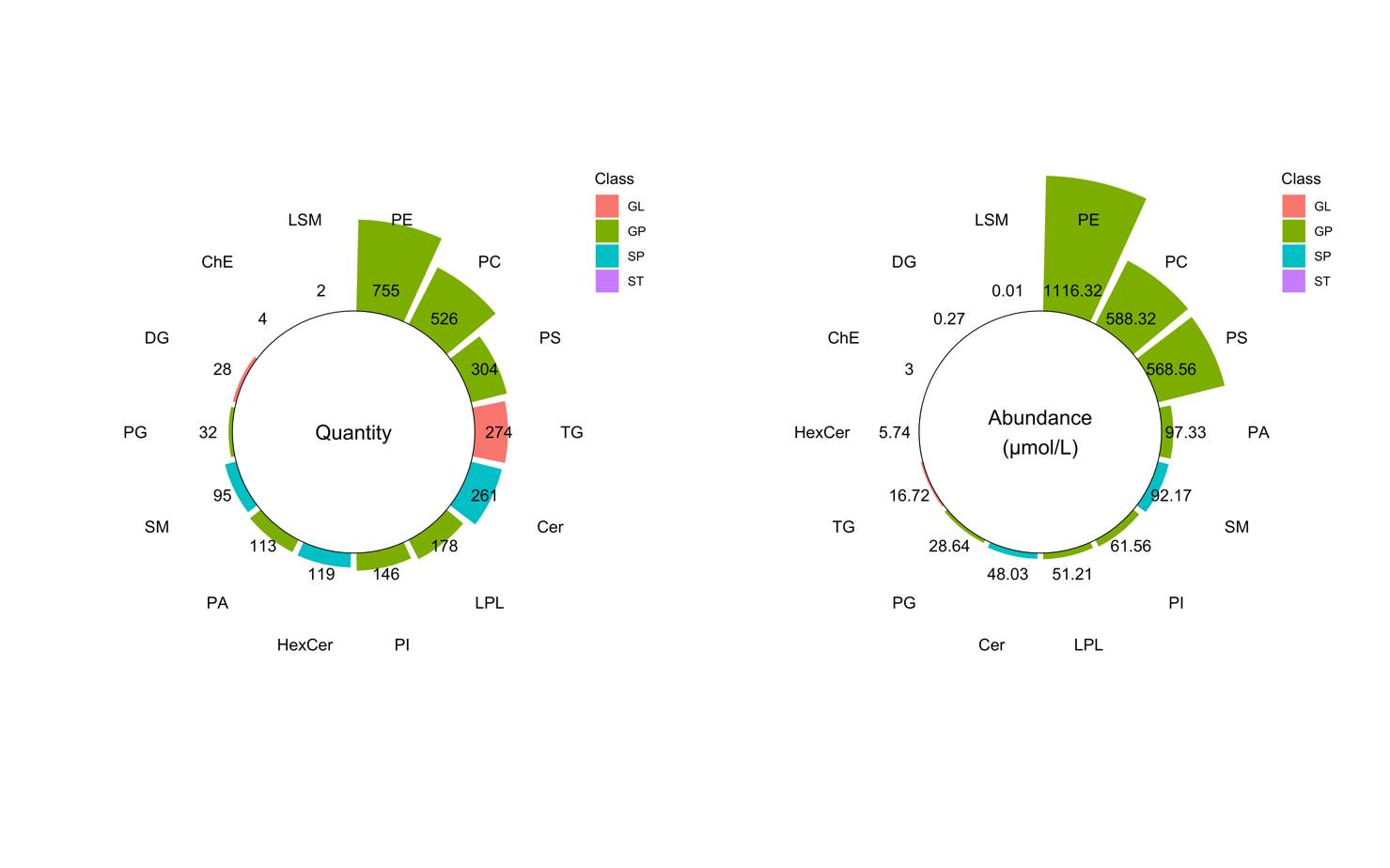
**

**Figure S4. The quantity and abundance of 2837 lipids in the erythrocyte membrane.** LPL, Lysophospholipid; PA, Phosphatidic Acid; PC, Phosphatidylcholine; PE, Phosphatidylethanolamine; PG, Phosphatidylglycerol; PI, Phosphatidylinositol; PS, Phosphatidylserine; DG, Diglyceride; TG, Triglyceride; Cer, Ceramide; HexCer, Hexosyl Ceramide; SM, Sphingomyelin; LSM, Lysosphingomyelin; ChE, Cholesterol Ester; GP, Glycerophospholipid; GL, Glycerolipid; SP, Sphingolipid; ST, Sterol Lipids.

**
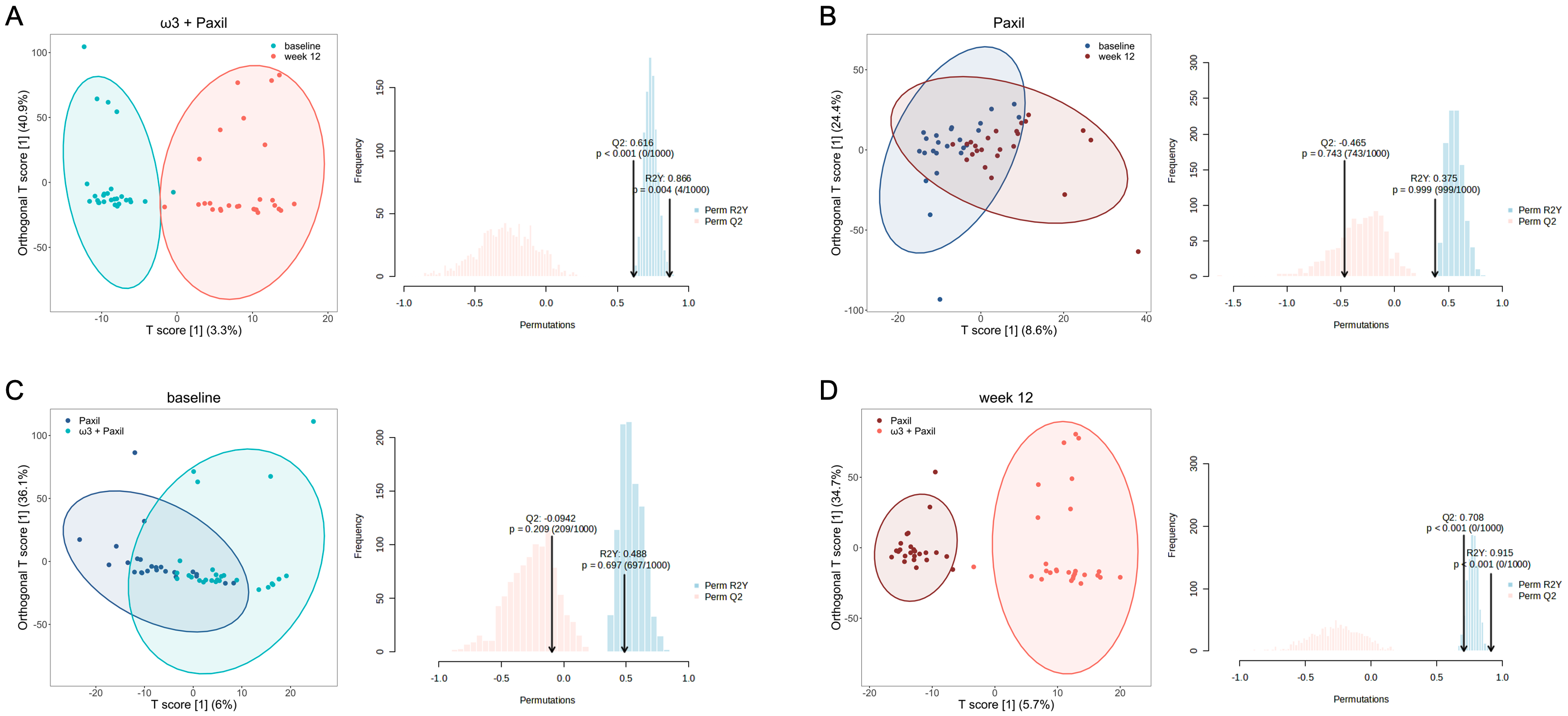
**

**Figure S5. Membrane lipids were obviously different after adjuvant ω3 PUFA supplementation.** OPLS-DA models of membrane lipidomic (A) between baseline and week 12 in ω3 + Paxil group, (B) between baseline and week 12 in Paxil group, (C) between ω3 + Paxil and Paxil group at baseline, (D) between ω3 + Paxil and Paxil group at week 12. ω3 PUFA, Omega-3 Polyunsaturated Fatty Acids. OPLS-DA, Orthogonal Partial Least Squares-Discriminant Analysis.


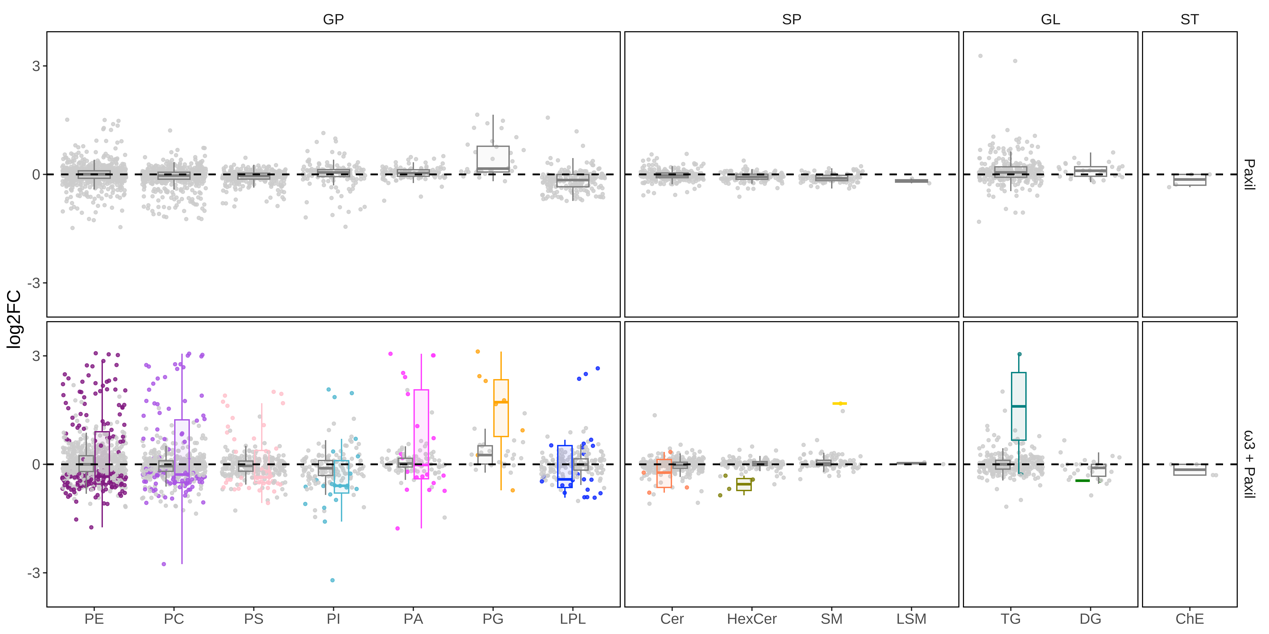


**Figure S6. The changes of membrane lipids between baseline and week 12 in ω3 + Paxil group or Paxil group.** Colored points are differential lipids, and gray points indicate no significant change in these lipids. FC, Fold Change; PE, Phosphatidylethanolamine; PC, Phosphatidylcholine; PS, Phosphatidylserine; PI, Phosphatidylinositol; PA, Phosphatidic Acid; PG, Phosphatidylglycerol; LPL, Lysophospholipid; Cer, Ceramide; HexCer, Hexosyl Ceramide; SM, Sphingomyelin; LSM, Lysosphingomyelin; TG, Triglyceride; DG, Diglyceride; ChE, Cholesterol Ester; GP, Glycerophospholipid; SP, Sphingolipid; GL, Glycerolipid; ST, Sterol Lipids.


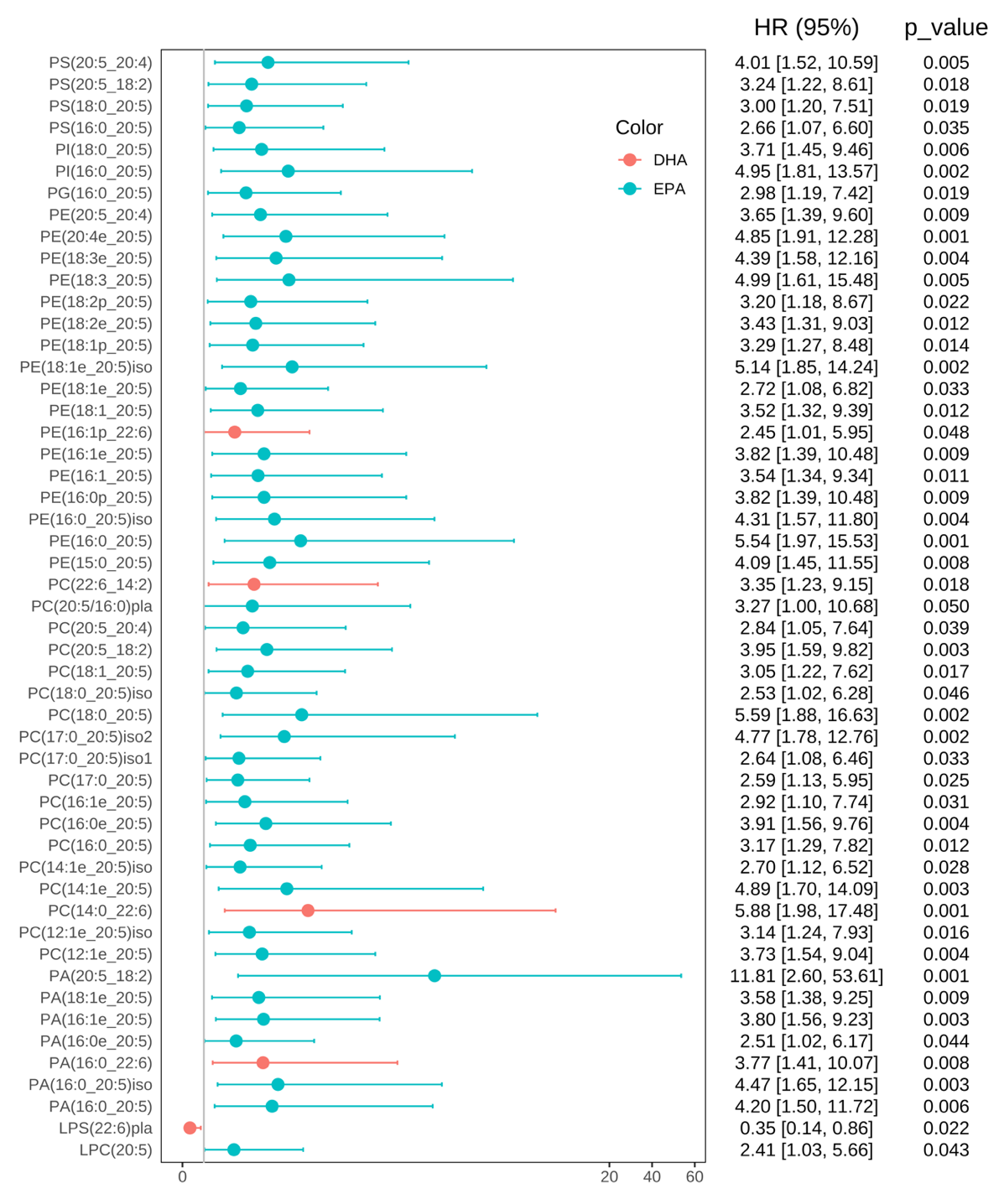


**Figure S7. Associations between changes of phospholipids levels containing EPA or DHA and clinical response.** The "pla" label indicates phospholipids in plasma, while the "iso" label refers to isomers on cell membrane phospholipids. EPA, Eicosapntemacnioc Acid; DHA, Docosahexaenoic Acid; HR, Hazard Ratios; PA, Phosphatidic Acid; PC, Phosphatidylcholine; PE, Phosphatidylethanolamine; PG, Phosphatidylglycerol; PI, Phosphatidylinositol; PS, Phosphatidylserine; LPC, Lysophosphatidylcholine; LPS, Lysophosphatidylserine.

**
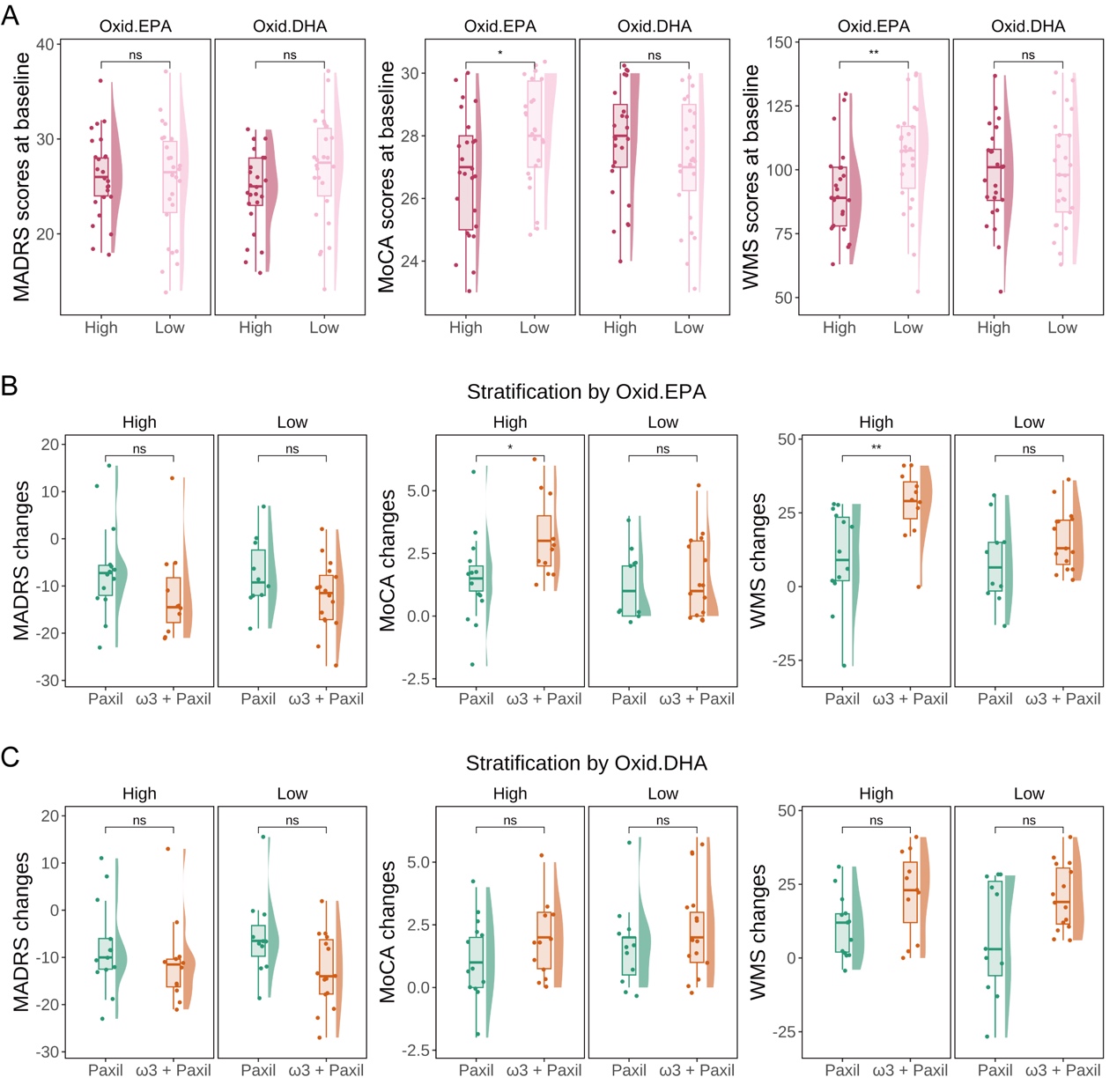
**

**Figure S8. ω3 PUFA was more effective in depressed adolescents with higher levels of oxidized EPA.** The differences of baseline MADRS, MoCA and WMS scores between subgroups with high and low (A) oxidized EPA levels or oxidized DHA levels. (B) Comparison of efficacy between ω3 + Paxil and Paxil group after stratification by oxidized EPA levels. (C) Comparison of efficacy between ω3 + Paxil and Paxil group after stratification by oxidized DHA levels. MADRS, Montgomery-Asberg Depression Rating Scale; MoCA, Montreal Cognitive Assessment; WMS, Wechsler Memory Scale; Oxid.EPA, Oxidized Eicosapntemacnioc Acid; Oxid.DHA, Oxidized Docosahexaenoic Acid; PUFA, Polyunsaturated Fatty Acids. *p<0.05, **p<0.01.
